# The development and initial findings of a DISGUST scale

**DOI:** 10.3389/fnhum.2025.1607506

**Published:** 2025-07-16

**Authors:** Tobias Herzl, Jürgen Fuchshuber, Sarah Straßnig, Afrodita Latifi, Peter Walla, Andreas Fink, Human-Friedrich Unterrainer

**Affiliations:** ^1^Institute of Psychology, University of Graz, Graz, Austria; ^2^Center for Integrative Addiction Research (CIAR), Grüner Kreis Association, Vienna, Austria; ^3^Department of Psychoanalysis and Psychotherapy, Medical University Vienna, Vienna, Austria; ^4^Comprehensive Center for Clinical Neurosciences and Mental Health, Medical University Vienna, Vienna, Austria; ^5^Department of Psychiatry and Psychotherapeutic Medicine, Medical University Graz, Graz, Austria; ^6^Faculty of Psychology, Sigmund Freud University Vienna, Vienna, Austria; ^7^Faculty of Medicine, Sigmund Freud University Vienna, Vienna, Austria; ^8^School of Psychology, Newcastle University, University Drive, Callaghan, NSW, Australia; ^9^Department of Religious Studies, University of Vienna, Vienna, Austria; ^10^Faculty of Psychotherapy Science, Sigmund Freud University Vienna, Vienna, Austria

**Keywords:** disgust, primary emotions, personality, psychopathology, scale development, affective neuroscience

## Abstract

**Background:**

Disgust is a fundamental emotion linked to survival, but its classification as a primary emotion remains debated. This study develops and validates a questionnaire assessing disgust as a primary emotion and examines its relationship with personality traits and psychopathology.

**Methods:**

A total of 482 German speaking participants completed an online survey. The sample was split for a principal component analysis (*N* = 250) and confirmatory (*N* = 232) factor analyses. Correlations and hierarchical regressions tested associations with personality traits and psychiatric symptoms.

**Results:**

Initial item reduction via PCA resulted in two alternative unidimensional models with eight and five Items. CFA confirmed excellent model fit for both versions (DISGUST-8: *χ2* = 13.00, *p* = 0.88, *df* = 20, *χ2/df* = 0.65, *RMSEA* = 0.000 (90% CI: 0.000, 0.057), *CFI* = 1.000; *NFI* = 0.992, *TLI* = 1.006, *SRMR* = 0.042; DISGUST-5: *χ2* = 0.893, *p* = 0.97, *df* = 5, *χ2/df* = 0.18, *RMSEA* = 0.000 (90% CI: 0.000, 0.092); *CFI* = 1.000; *NFI* = 0.999; *TLI* = 1.011; *SRMR* = 0.017). Internal consistency was high for both versions (DISGUST-8: *α* = 0.89; DISGUST-5: *α* = 0.88). Trait disgust correlated highest with neuroticism (*r_DISGUST-8_* = 0.36; *r_DISGUST-5_* = 0.36) and anxiety (*r_DISGUST-8_* = 0.27; *r_DISGUST-5_* = 0.28). Regression analysis confirmed disgust as a significant predictor of neuroticism (*t*(457) = 4.19, *β* = 0.12).

**Discussion:**

The findings highlight disgust’s role in personality and psychopathology. The developed scale reliably measures disgust, demonstrating its association with neuroticism. Future research should explore cross-cultural validation and refine the scale’s clinical applicability.

## Introduction

Emotions play a fundamental role in human experience and behavior, shaping perception, decision-making, and social interactions. Over the last decades several primary emotion frameworks have been developed, e.g., by Ekman (1999), Izard (1992), and Krause (2012). In contrast to the authors mentioned above, Panksepp’s (1998) primary emotion concept is rooted in biological psychology. He considers primary emotions as evolutionary developed processes and innate mechanisms that regulate adaptive responses and are hardwired within the mammalian brain (Panksepp, 2011). While Panksepp’s (1998) classical model identifies seven primary emotions, the classification of DISGUST remains controversially discussed (Panksepp, 2007; Toronchuk and Ellis, 2007a, 2007b; Tolchinsky et al., 2024). This study aims to contribute to this debate by developing and validating a questionnaire to assess DISGUST in context of the primary emotion framework. Additionally, it examines the influence of DISGUST on personality and well-being, providing new insights into its psychological and affective significance.

### Primary emotions

The concept of primary emotions, as described by Panksepp (1998), originates from animal research but has been increasingly applied to humans in recent years (Marengo et al., 2021; Brienza et al., 2023). Panksepp (2011) argues that these emotions are evolutionarily embedded, innate mechanisms that govern behavior and are essential for survival.

Panksepp (1998) identified seven primary emotions (SEEKING, CARE, PLAY, LUST, ANGER, FEAR, SADNESS) that are anchored in specific neural networks of the brain and influence our cognitive and affective processes based on a bottom-up principle. A key characteristic of these emotions is that they can be experimentally triggered through targeted electrical stimulation of specific brain regions (Panksepp, 2011). Another characteristic is that primary emotions are linked to our personality, specifically to the Big Five personality traits. According to the authors, SEEKING is associated with openness, PLAY with extraversion, CARE/ANGER with agreeableness, and SADNESS/FEAR/ANGER with neuroticism (Montag and Panksepp, 2017).

Generally, a distinction is made between positive/pleasurable and negative/unpleasurable emotional systems (Kernberg, 2001, 2022). SEEKING, CARE, PLAY, and LUST are on the positive side of the affective spectrum. For instance SEEKING drives mammals to find vital resources such as food and mating partners. CARE plays a central role in nurturing and raising offspring, ensuring their survival. PLAY fosters both motoric and social development and strengthens bonds. LUST is equally essential, as it drives reproduction and ensures the survival of the species (Panksepp, 2011).

In contrast, SADNESS, ANGER, and FEAR can be grouped together to the negative affective spectrum: SADNESS protects against social isolation and manifests as separation anxiety, grief and sadness, while ANGER serves to defend offspring and territory through aggressive behavior. The FEAR system enables mammals to respond to threats by activating flight or freeze mechanisms (Panksepp, 2011).

### Disgust

The primary reason why both humans and animals experience disgust are to avoid diseases by preventing foreign objects or pathogens from entering the organism, thereby ensuring survival. In its original form, disgust prevents the ingestion of disease-causing pathogens (viruses, bacteria, parasites) through the mouth (Rozin et al., 2008; Chapman and Anderson, 2012).

It is important to clearly distinguish disgust from the concept of distaste. Distaste is a sensory affect triggered by taste (especially bitter substances) that causes a reflex leading to spitting out ingested material. Its function is assumed to be the avoidance of toxins. Disgust, on the other hand, is more complex, as it primarily prevents objects from being ingested in the first place, which is referred to in literature as core disgust (Rozin et al., 2008; Chapman and Anderson, 2012). Therefore, an organism must rely on certain stimuli that reliably indicate the presence of pathogens. These stimuli can be olfactory, tactile or visual, such as slimy substances, feces, insects or the smell of decay. They can also include objects that are likely to transmit pathogens, such as a glass from which a person with a cold sore has drunk.

Disgust has evolved over time, moving beyond ingestion to continue to prevent disease and ensure survival. Chapman and Anderson (2012) group this under physical disgust, which includes core disgust as well as blood–injury, interpersonal, and sexual disgust. Blood–injury disgust refers to disgust towards injuries and blood, while interpersonal disgust refers to disgust towards dead or unfamiliar living beings. Sexual disgust relates to sexual contact with the wrong species or with very old or very young beings.

Through socialization, cultural, and moral norms, humans have also developed what is known as moral disgust. Moral disgust is less about avoiding pathogens and more about behaviors that are culturally unacceptable (e.g., rape, cannibalism) (Marzillier and Davey, 2004; Rozin et al., 2008; Chapman and Anderson, 2012).

Experiencing disgust naturally leads to specific bodily reactions and behaviors. The most widespread universal and cross-cultural reaction is the disgust-specific facial expression, characterized by furrowing of the eyebrows and nose, opening of the mouth, and downward pulling of the mouth corners (Izard, 1971; Ekman and Friesen, 1978; Darwin, 1998). It is believed that this facial expression serves as a protective mechanism to minimize the risk of pathogens entering the body (Susskind et al., 2008). However, Toronchuk and Ellis (2007a) as well as Panksepp (2007) have questioned whether this expression can be considered a valid predictor of disgust, as it is difficult to distinguish from the expression triggered by distaste. Other reactions include feelings of nausea or revulsion (Rozin et al., 2008), avoidance of objects perceived as “disgusting” (Woody and Teachman, 2000), and a decrease in heart rate, which contrasts with an increase in heart rate observed in fear (Ekman et al., 1983; Stark et al., 2005), and may even lead to dizziness or fainting (Chapman and Anderson, 2012).

### Neuropsychological foundations

Neural activity related to disgust has been extensively studied in the literature, with the insula emerging as a key brain region. Baumann and Mattingley (2012) found that only anger and disgust activate the insula in the left hemisphere suggesting overlapping neural networks of both emotions. Wright et al. (2004) also found that the anterior insula is activated, especially when viewing images depicting injuries, diseases, or unsanitary scenes (e.g., garbage, mold). A meta-analysis by Gan et al. (2022) confirmed that the anterior insula’s role in processing disgust and additionally identified that the inferior frontal gyrus and fusiform gyrus play a role in processing disgust-inducing stimuli. Furthermore, lesions in the insula have been shown to impair disgust processing (Gan et al., 2022).

The anterior insula is also activated when viewing the characteristic facial expression of disgust (Chapman and Anderson, 2012) and electrical stimulation studies have produced similar facial reactions in monkeys, by stimulation of the anterior insula (Caruana et al., 2011). Moreover electrophysical stimulation studies were also able to demonstrate the role of the insula in human disgust recognition (Papagno et al., 2016). However, targeted stimulation of the disgust emotion is challenging, specifically, as the brain regions involved in processing disgust overlap with other affective systems, particularly FEAR, SADNESS, ANGER and SEEKING (Toronchuk and Ellis, 2007a). In contrast to a strict localist understanding of the brain, current neurophysiological models of disgust assume concerted activity of brain wide networks including subcortical regions like the amygdala, PAG, thalamus and putamen (tasked with threat detection and avoidance behavior) and cortical areas like anterior insula, dorsal anterior cingulate cortex and default mode network, which are engaged in interoception and affect assessment, as well as regions associated with bitter taste detection (posterior insula and brain stem) (Vicario et al., 2017; Gan et al., 2022; Gan et al., 2024).

### DISGUST as a primary emotion

According to Panksepp (2007), seven criteria must be met for an emotion to be considered a primary emotion or primary emotional system: (1) The emotion should be activated by unconditioned environmental stimuli, meaning it should not be a mere reflex response; (2) It should trigger a coherent sequence of behaviors; (3) The system should be capable of processing environmental information; (4) The emotional activity should persist even after the triggering stimulus has ceased; (5) Cognitive processes should elicit emotional responses; (6) The emotion should activate and regulate complex cognitive strategies; and (7) primary emotions should be capable of generating and explaining psychiatric disorders.

The findings presented above suggest that, despite Panksepp (2007) criticism, DISGUST can be considered a primary emotion, particularly core disgust (Toronchuk and Ellis, 2007a, 2007b) and Tolchinsky et al. (2024) also argue convincingly in their papers why DISGUST should be considered a primary emotion. Tolchinsky et al. (2024) emphasize that disgust is a flexible emotional system that extends beyond simple oral distaste, functioning to protect the internal milieu from pathogens through a complex interplay of neural, cognitive, and immune-related processes. Their study builds upon previous work by Toronchuk and Ellis (2007b), demonstrating that disgust involves distinct neural circuits and resulting behaviors are more complex than simple reflexes (Toronchuk and Ellis, 2007a).

Another argument supporting this is the link between disgust and the immune system. For instance women exhibit heightened disgust sensitivity during the second half of the menstrual cycle (luteal phase) due to a reduced inflammatory immune response, which makes them more susceptible to infections (Fleischman and Fessler, 2011). Similarly, increased disgust sensitivity is observed during pregnancy to enhance the newborn’s survival chances (Fessler et al., 2005). Tolchinsky et al. (2024) further highlights that disgust acts as an affective partner to the immune system, with behavioral and physiological responses working in tandem to minimize pathogen exposure and maintain internal stability.

Further support for classifying DISGUST as a primary emotion comes from research by Siegel et al. (2009), which demonstrates disgust’s ability to process environmental information, one of the key features of primary emotional systems, according to Panksepp (2007). In two experiments, the authors showed that disgust-related stimuli, such as tools covered with a repellent substance, not only altered participants grasping behavior but also significantly influenced their perception of distance to the objects (Siegel et al., 2009). These findings suggest that disgust-related cues are detected, evaluated, and incorporated into sensorimotor planning, supporting the view that DISGUST processes information from the environment and guides adaptive behavioral responses.

A significant point of criticism on Panksepp (2007) was that he did not attribute psychiatric relevance to DISGUST. However, studies have shown that disgust is associated with several psychiatric disorders, particularly Obsessive-Compulsive Disorder (OCD) with contamination fear (Olatunji et al., 2008; Davey, 2011; Tolchinsky et al., 2024). There is even initial evidence that disgust proneness (i.e., the likelihood and intensity of experiencing disgust) could be both a result and a cause of OCD (Olatunji and Kim, 2024). Therefore interventions in disgust dysregulation may be crucial for treating post-traumatic OCD with contamination obsessions, as well as for conditions like emetophobia and hypochondriasis (Tolchinsky et al., 2024). Further psychiatric relevance is demonstrated by a meta-analysis by Mitchell et al. (2024), which found that disgust is more resistant to extinction than fear. This could be relevant for the treatment of individuals with arachnophobia, as disgust is suspected to be the primary emotion underlying this disorder (Davey, 2011). Disgust also appears to play a significant role in post-traumatic stress disorders (PTSD), particularly in relation to sexual trauma. Women tend to feel increased disgust towards themselves after sexual assaults, suggesting that PTSD is often accompanied by heightened feelings of disgust (Badour and Feldner, 2018).

Finally, another aspect that supports the classification of DISGUST as a primary emotion, according to Panksepp (2007), is its association with personality traits. Numerous studies have demonstrated such associations, although the direction of the relationships is not always clear. Druschel and Sherman (1999) found a positive correlation between disgust and agreeableness, whereas more recent studies suggest a negative relationship (Tybur et al., 2009; Tybur and De Vries, 2013; Kupfer and Tybur, 2017). Olatunji et al. (2008) found no association with core disgust. The relationship between disgust and extraversion also appears unclear, as Gangestad and Grebe (2014) found a positive correlation, while other studies found no relationship (Olatunji et al., 2008; Tybur et al., 2009; Tybur and De Vries, 2013; Kupfer and Tybur, 2017). Similar inconsistencies have been observed with openness and conscientiousness, whereas neuroticism consistently shows a positive association with disgust (Olatunji et al., 2008; Tybur et al., 2009; Tybur and De Vries, 2013; Kupfer and Tybur, 2017).

For these reasons, it is assumed that DISGUST can indeed be considered a primary emotion. However, this view is not universally accepted. From a psychological constructionist perspective, emotions are not biologically hardwired entities but are constructed from more general processes such as bodily sensations, conceptual knowledge, and situational context (Lindquist et al., 2013). Supporting this view, a meta-analysis by Siegel et al. (2018) found high variability in autonomic responses across and within emotion categories, including disgust, challenging the idea of distinct physiological emotion “fingerprints.” Despite such variability, the present study adopts a neurofunctional framework that conceptualizes DISGUST as a primary emotion. Converging evidence from behavioral research, neuroscience, immunological patterns and psychiatric as well as personality correlates underscores the consistency and adaptive function of DISGUST across contexts.

This study builds upon that theoretical foundation by developing a questionnaire to assess DISGSUT within the affective neuroscience framework and examines the role of DISGUST in influencing human personality and well-being. While there are numerous questionnaires that assess various constructs of disgust (e.g., disgust proneness: DES, Olatunji et al., 2015; disgust propensity: DS-R, van Overveld et al., 2011), none to date have focused on DISGUST in the construct of affective neuroscience. Moreover, existing questionnaires tend to be relatively long. In particular, there are no short questionnaires in the German-speaking context that exclusively assess core disgust. This research thus aims to close that gap by developing a psychometrically sound and more economical questionnaire and also explores how DISGUST relates to psychiatric disorders and whether DISGUST, in addition to the existing primary emotions, can influence aspects of personality.

## Materials and methods

### Sample and procedure

In the first step, an item pool consisting of 36 questions was developed, based on extensive literature research, to capture DISGUST as a primary emotion. Subsequently, a test battery was created, which included demographic data as well as psychological self-assessment questionnaires. The socio-demographic data comprised questions on gender, sex, age, marital status, children, level of education, field of study, current occupation, sexual orientation, country of origin, language, current psychiatric disorders, medication, religion, and spirituality. Data collection was conducted online using LimeSurvey^©^ from July to October 2024, with the survey taking approximately 40 min to complete. Recruitment was carried out via an online link distributed through flyers, social media platforms (Facebook, Instagram, WhatsApp, etc.), mailing lists, and various forums. Eligibility criteria for participation included completing an informed consent form prior to the survey, being of legal age, and having fluent German language skills. Participants were fully anonymized and could terminate the survey at any time without providing reasons. Among participants who completed the survey, vouchers were raffled. The study was approved by the Ethics Committee of the University of Graz (Nr. 183–2023/24).

### Sample characteristics

**Figure 1:**
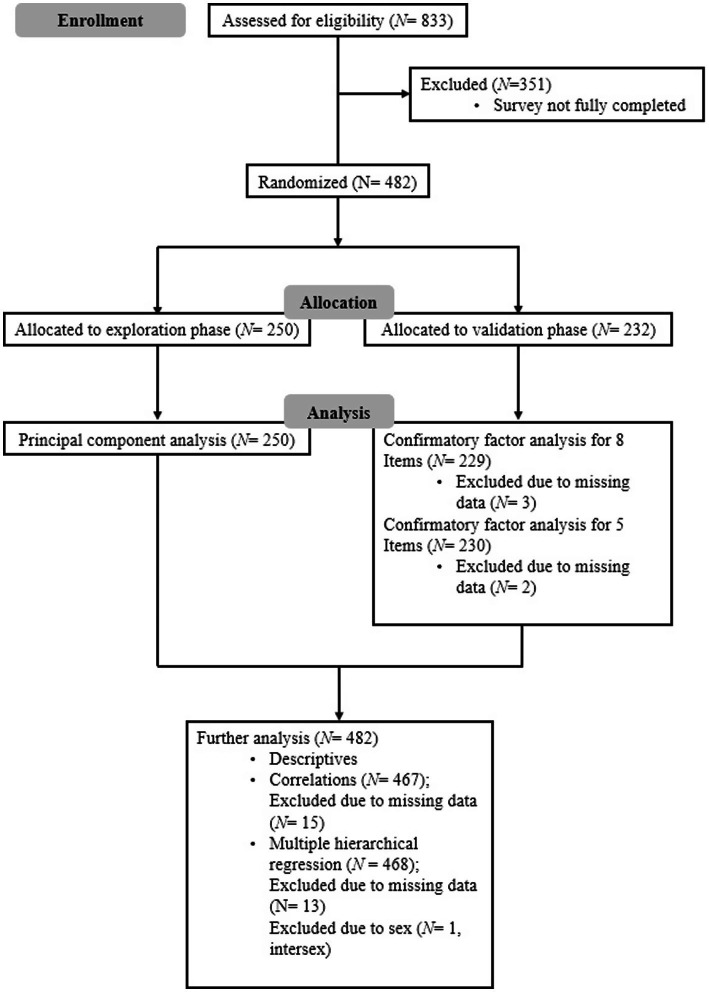
Flowchart of data assessment.

A total of 833 individuals were reached, of whom 482 fully completed the necessary questionnaires for the study. Among these, 147 participants identified as male, and 334 identified as female, while one participant identified as intersex. The average age was 40.4 years (*SD* = 18.323), with the youngest participant being 18 years old and the oldest 86 years old. Of the participants, 451 (93.6%) were from Austria, Germany, or Switzerland. The remaining participants were from another EU country (4.8%) or a non-EU country (1.7%). 35.7% reported having a high school diploma as their highest educational qualification, while 24.9% held a master’s degree. The largest group comprised students (33.8%), followed by employees, workers, and civil servants (29.9%). Among those who had attended or were attending a university, psychology students represented the largest group (12.7%). At the time of the study, 37 participants (7.7%) reported having a diagnosed mental illness. The self-reported diagnoses included depression (*N* = 18), post-traumatic stress disorder (*N* = 9), attention deficit hyperactivity disorder (*N* = 3), autism spectrum disorder (*N* = 3), bipolar disorder type I or II (*N* = 3), adjustment disorder (*N* = 3), borderline personality disorder (*N* = 2), anxiety disorder (*N* = 2), anorexia nervosa (*N* = 1), obsessive-compulsive disorder (*N* = 1), dissociative seizures (*N* = 1), impulse control disorder (*N* = 1) and insomnia (*N* = 1). Characteristics for the exploratory and validation groups are presented in Table 1. Further information can be seen in Figure 1.

**Table 1:** Sample characteristics for exploration and validation phase.

Sample	Exploration phase	Validation phase	
Overall	*N =* 250	*N =* 232	
Sex	*N =* 72 male (28.8%)	*N =* 75 male (32.2%)	
	*N =* 177 female (70.8%)	*N =* 157 female (67.7%)	
	*N =* 1 intersex (0.4%)		
Age	*M =* 40.48 years; *SD =* 18.467	*M =* 40.3 years; *SD =* 18.210	
Nationality	*N =* 232 DACH-region (92.8%)	*N =* 219 DACH-region (94.4%)	
	*N =* 14 other EU country (5.6%)	*N =* 9 other EU country (3.9%)	
	*N =* 4 non EU country (1.6%)	*N =* 4 non EU country (1.7%)	
Highest level of education	*N =* 3 compulsory school (1.2%)	*N =* 4 compulsory school (1.7%)	
	*N =* 40 vocational training (16%)	*N =* 20 vocational training (8.6%)	
	*N =* 85 A-levels (34%)	*N =* 87 A-levels (37.5%)	
	*N =* 42 Bachelor (16.8%)	*N =* 36 Bachelor (15.5%)	
	*N =* 62 Master (24.8%)	*N =* 85 Master (25%)	
	*N =* 7 Doctorate (2.8%)	*N =* 15 Doctorate (6.5%)	
	*N =* 10 other university degree (4%)	*N =* 12 other university degree (5.2%)	
	*N =* 1 no qualification (0.4%)		
Field of study	*N =* 33 Psychology (13.2%)	*N =* 27 Psychology (11.6%)	
	*N =* 6 Human/Veterinary Medicine and Dentistry (2.4%)	*N =* 9 Human/Veterinary Medicine and Dentistry (3.9%)	
	*N =* 3 Health Sciences (1.2%)	*N =* 6 Health Sciences (2.6%)	
	*N =* 5 Computer Science and Information Technology (2%)	*N =* 1 Computer Science and Information Technology (0.4%)	
	*N =* 13 Engineering (5.2%)	*N =* 6 Engineering (2.6%)	
	*N =* 19 Natural Science (7.6%)	*N =* 21 Natural Science (9.1%)	
	*N =* 29 Humanities (11.6%)	*N =* 23 Humanities (9.9%)	
	*N =* 7 Law (2.8%)	*N =* 7 Law (3%)	
	*N =* 2 Religious studies (0.8%)	*N =* 8 Religious studies (3.4%)	
	*N =* 15 Social Science (6%)	*N =* 13 Social Science (5.6%)	
	*N =* 10 Economics (4%)	*N =* 12 Economics (5.2%)	
	*N =* 29 Teaching (11.6%)	*N =* 41 Teaching (17.7%)	
	*N =* 28 other (11.2%)	*N =* 23 other (9.9%)	
	*N =* 51 No data (20.4%)	*N =* 35 No data (15.1%)	
Occupation	*N =* 2 school (0.8%)	*N =* 9 school (3.9%)	
	*N =* 1 apprenticeship (0.4%)	*N =* 5 apprenticeship (2.2%)	
	*N =* 83 studies (33.2%)	*N =* 80 studies (34.5%)	
	*N =* 71 Employee, Worker, Civil servant (28.4%)	*N =* 73 Employee, Worker, Civil servant (31.5%)	
	*N =* 36 self-employed (14.4%)	*N =* 23 self-employed (9.9%)	
	*N =* 6 parental leave (2.4%)	*N =* 3 parental leave (1.3%)	
	*N =* 42 retirement (16.8%)	*N =* 33 retirement (14.2%)	
	*N =* 1 unemployed (0.4%)	*N =* 3 unemployed (1.3%)	
	*N =* 8 part-time job (3.2%)	*N =* 3 part-time job (1.3%)	
Diagnosed psychiatric disorder	*N =* 20 (8%)	*N =* 17 (7.3%)	

M, mean; N, population; SD, standard deviation; DACH-region: Germany, Austria, Switzerland.

### Item generation

Through extensive literature research, 36 questions were developed to capture DISGUST as a primary emotion. These questions were designed to address the disgust-specific factors identified by Chapman and Anderson (2012): (1) core, (2) interpersonal, and (3) blood–injury disgust. These factors, summarized under the category of physical disgust, encompass olfactory, tactile, visual, and auditory elements. They reflect the essence of the primary emotion DISGUST: avoiding infections to ensure survival. Additionally, the questions aimed to represent typical reactions triggered by disgust (Davey, 2011; Chapman and Anderson, 2012).

Particular attention was paid to formulating the questions in a culturally inclusive manner. To achieve this, overlaps of potential disgust stimuli across cultures were identified. This approach was based on a study by Curtis and Biran (2001), which captured disgust stimuli across various cultures (UK, Netherlands, Africa, India, international airports). Examples include (body) secretions, excretions, body parts, certain animals, spoiled or decaying substances and dirty toilets.

The questions followed the format of the Brief Affective Neuroscience Personality Scale (BANPS-GL), as DISGUST was intended to serve as an additional scale for this questionnaire. The response format was thus a 5-point Likert scale with the following options: (1) strongly disagree, (2) disagree, (3) neither agree nor disagree, (4) agree, (5) strongly agree.

### Data analysis

The data were analyzed using IBM SPSS Statistics (Version 29) and RStudio 2024.12.0 + 467. SPSS was used to calculate descriptive statistics and correlations. Additionally, SPSS was employed for multiple hierarchical regressions and principal component analysis (PCA) to identify the factor structure and to examine the extent to which DISGUST can associate personality traits. In a subsequent step, confirmatory factor analysis (CFA) was conducted using RStudio to verify whether DISGUST could be considered a unidimensional model. The Weighted Least Squares Mean and Variance Adjusted (WLSMV) was used as estimator, as it is robust to potential violations of normal distribution and is better suited for the use with ordinally scaled items (DiStefano and Morgan, 2014).

Correlations, regressions, and descriptive statistics were computed for the entire sample (*N* = 482). To verify the validity of the questionnaire, Pearson correlations were calculated between the mean scores of the long and short versions of the DISGUST scale and the scales of the Big Five Inventory – Short Version (BFI-K), ICD-10 Symptom Rating (ISR), and BANPS-GL. Multiple hierarchical regression was performed using the ENTER method, with sex and age as control variables.

For further analysis, the sample was randomized and divided into two parts. Sample A (*N* = 250) was used to identify the factor structure via PCA. Sample B (*N* = 232) was utilized for CFA. The following fit indices were defined to signal an acceptable fit: (a) The comparative fit index (CFI) > 0.90; (b) Tucker-Lewis index (TLI) > 0.90; (c) The root mean square error of approximation (RMSEA) < 0.08, with the upper bound of its 90% confidence interval < 0.1 (Kline, 2023).

### Item reduction

Sample A was used to test the initial 36 items. The initial goal was to create a long and short version of the DISGUST scale based on the format of the existing BANPS-GL scales. The reduction process focused on ensuring (1) the scale’s reliability with Cronbach’s *α* > 0.8 (Blanz, 2021), (2) discriminatory power with *r_it_* > 0.3, (3) item difficulty primarily being moderate, with some easy and difficult items, and (4) unidimensionality through principal component analysis. The iterative reduction process ultimately resulted in an 8-item version (DISGUST-8) and a 5-item version (DISGUST-5).

### Psychometric assessments

#### Brief affective neuroscience personality scale (BANPS-GL)

The BANPS-GL is the German version designed to capture the six primary emotions PLAY, ANGER, SEEK, CARE, FEAR, and SADNESS. It includes 33 questions, with responses given on a 5-point Likert scale ranging from (1) strongly disagree to (5) strongly agree. The questionnaire demonstrates acceptable internal reliability across all scales (*ω_CARE_* = 0.70, *ω_SEEK_* = 0.72, *ω_PLAY_* = 0.76, *ω_FEAR_* = 0.84, *ω_ANGER_* = 0.80, *ω_SADNESS_* = 0.86) (Fuchshuber et al., 2023). Additionally, the B-ANPS-L was administered to capture the primary emotion LUST using 21 items. For the calculations in this study, however, the short version with 5 items, which has a reliability of McDonald’s *ω* = 0.79, was used (Fuchshuber et al., 2023). Finally, the 36 questions on DISGUST were included. See the item generation section for details.

#### Big five inventory – short version (BFI-K)

The BFI-K is a self-assessment questionnaire and the German short version of the Big Five Inventory. It consists of 21 items with an average completion time of 2 min. It captures the five personality traits Extraversion, Agreeableness, Conscientiousness, Neuroticism, and Openness on a 5-point Likert scale, where (1) “very inaccurate” and (5) “very accurate.” Although all scales yielded lower internal consistencies compared to the standard version, the alpha coefficients in all cases exceeded the values estimated by the Spearman-Brown formula for shortened tests (*α_Extraversion_* = 0.81, α_est_ = 0.75; *α_Agreeableness_* = 0.59, *α_est_* = 0.53; *α_Conscientiousness_* = 0.69, α_est_ = 0.64; *α_Neuroticism_* = 0.77, *α_est_* = 0.67; *α_Openness_* = 0.70, *α_est_* = 0.60). Additionally, the scales demonstrate high average stability of *r_tt_* = 0.84 (Rammstedt and John, 2005).

#### ICD-10 symptom rating (ISR)

The ISR is a screening questionnaire designed to capture psychiatric symptoms and their severity. It consists of 29 items across six scales (Depression, Anxiety, Compulsion, Somatization, Eating Disorder, Supplementary Items). Each item can be answered on a 5-point Likert scale ranging from (0) “does not apply” to (4) “applies extremely.” The questionnaire was cross-validated with the SCL-90-R and achieved correlations between syndrome scales ranging from *r* = 0.37 to *r* = 0.78 (Tritt et al., 2008). Test–retest reliability is satisfactory, ranging from *r_tt_* = 0.70 to *r_tt_* = 0.94 (Fischer et al., 2011).

## Results

### Principal component analysis

PCA was conducted to determine the dimensional structure of the model and to extract the main factors. The items were suitable for PCA as indicated by a Kaiser-Meyer-Olkin criterion *KMO* = 0.919 and a significant Bartlett’s Test of Sphericity (*Chi-Square* (630) = 4208.122, *p* < 0.001). The scree plot (can be found in the Supplementary file) and the factor solution suggested a unidimensional model. This was also evident from 32.746% total variance explained (eigenvalue = 11.788). Factor loadings ranged from 0.319 to 0.784. Based on previous considerations (see item reduction) the factor “DISGUST-8” (factor loadings from 0.625 to 0.863; eigenvalue = 4.499 (56.234%)) was created with 8 items. Further reduction resulted in the creation of the factor “DISGUST-5” (factor loadings from 0.629 to 0.903; eigenvalue = 3.418 (68.354%)) with 5 items. Detailed factor loadings can be found in Table 2.

**Table 2:** Factor loadings of DISGUST-8 and DISGUST-5.

Item	DISGUST-8	DISGUST-5
EK16	0.797	0.785
EK17	0.863	0.903
EK27	0.628	0.629
EK33	0.848	0.894
EK35	0.860	0.890
EK11	0.681	
EK15	0.642	
EK29	0.625	

The precise phrasing of each item, along with their English translation, is provided in the Supplementary file.

Additionally, skewness and kurtosis were analyzed to assess the distribution of the data. For DISGUST-8, skewness was 0.241 (*SD* = 0.113) and kurtosis was −0.358 (*SD* = 0.225), indicating a normal distribution. For DISGUST-5, skewness was 0.347 (*SD* = 0.113) and kurtosis was −0.469 (*SD* = 0.225), indicating slight non-normality. Further item properties are presented in Table 3.

**Table 3:** Item characteristics for DISGUST 5 and 8 item version.

Item	Mean	Standard deviation	Variance	Discriminant power	Item difficulty
EK16	2.91	1.130	1.276	0.689	47.75
EK17	2.54	1.145	1.310	0.804	38.5
EK27	3.01	1.195	1.429	0.533	50.25
EK33	2.46	1.160	1.345	0.799	36.5
EK35	2.13	1.086	1.180	0.775	28.25
EK11	2.49	1.086	1.180	0.625	37.25
EK15	3.63	1.003	1.006	0.553	65.75
EK29	2.61	1.238	1.533	0.539	40.25

DISGUST-8 consists of: EK27, EK33, EK35, EK16, EK17, EK29, EK15, EK11; DISGUST-5 consists of: EK27, EK33, EK35, EK16, EK17; The precise phrasing of each item, along with their English translation, is provided in the Supplementary file.

### Confirmatory factor analysis

**Figure 2:**
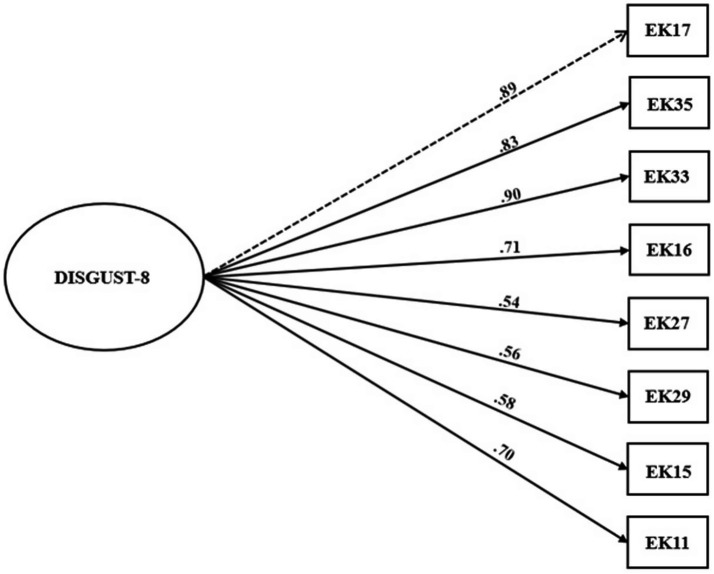
Model of DISGUST-8 scale*. N* = 229.

**Figure 3:**
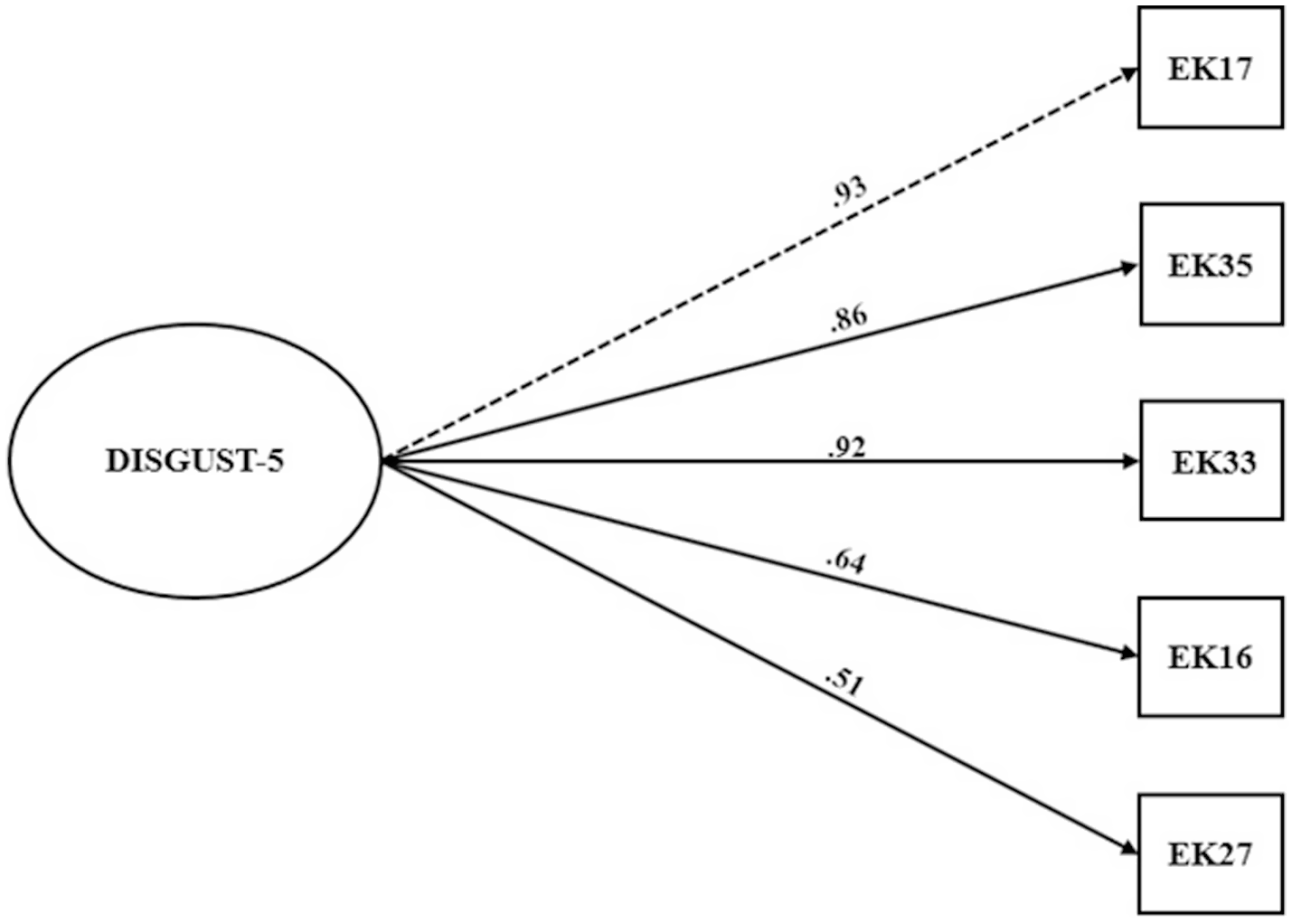
Model of DISGUST-5 scale. *N* = 230.

CFA (estimator: WLSMV) was carried out using Sample B. Both the DISGUST-8 and DISGUST-5 versions demonstrated excellent fit (DISGUST-8: *χ2* = 13.00, *p* = 0.88, *df* = 20, *χ2/df* = 0.65, *RMSEA* = 0.000 (90% CI: 0.000, 0.057), *CFI* = 1.000; *NFI* = 0.992, *TLI* = 1.006, *SRMR* = 0.042; DISGUST-5: *χ2* = 0.893, *p* = 0.97, *df* = 5, *χ2/df* = 0.18, *RMSEA* = 0.000 (90% CI: 0.000, 0.092); *CFI* = 1.000; *NFI* = 0.999; *TLI* = 1.011; *SRMR* = 0.017). The models are illustrated in Figures 2, 3.

### Reliability and validity

Cronbach’s α and McDonald’s *ω* total were used as a measure of internal consistency. Regarding Cronbach’s α both scales achieved high internal consistency (*α_DISGUST-8_* = 0.888; *α_DISGUST-5_* = 0.877; *N* = 472) according to Blanz (2021). This also holds true for McDonald’s ω, showing excellent internal consistency for DISGUST-8 (*ω_t_* = 0.913) and DISGUST-5 (*ω_t_* = 0.904).

Both scales correlated almost identically with those of the BFI-K, ISR, and BANPS-GL. All correlations were weak to moderately significant, except for DISGUST-8 and DISGUST-5 x Conscientiousness and DISGUST-8 and DISGUST-5 x Openness to Experience.

Regarding the Big Five personality traits, both the long and short versions correlated highest with Neuroticism (*r_DISGUST-8_* = 0.357, *p* < 0.001; *r_DISGUST-5_* = 0.363, *p* < 0.001).

Regarding psychiatric symptoms, the Anxiety scale correlated highest with DISGUST-8 (*r* = 0.272, *p* < 0.001) and DISGUST-5 (*r* = 0.279, *p* < 0.001), followed by the ISR sum score (*r_DISGUST-8_* = 0.253, *p* < 0.001; *r_DISGUST-5_* = 0.255, *p* < 0.001).

Furthermore, both DISGUST versions correlated weakly to moderately with the six primary emotions. The strongest correlation was found with FEAR (*r_DISGUST-8_* = 0.310, *p* < 0.001; *r_DISGUST-5_* = 0.287, *p* < 0.001). Detailed results are presented in Table 4.

**Table 4:** Pearson-Correlation (two tailed) between DISGUST 5 and 8 item version and BFI, ISR, and BANPS-GL.

Parameter	DISGUST-8	DISGUST-5
Extraversion	−0.170 **	−0.189 **
Agreeableness	−0.225 **	−0.210 **
Conscientiousness	−0.004	−0.016
Neuroticism	0.357**	0.363 **
Openness to experience	−0.015	−0.022
Depression	0.175 **	0.179 **
Anxiety	0.272 **	0.279 **
Compulsion	0.137 *	0.133 **
Somatization	0.164 **	0.167 **
Eating disorder	0.219 **	0.213 **
Supplementary items (ISR)	0.133**	0.136**
Sum score (ISR)	0.253 **	0.255 **
PLAY	−0.098 *	−0.103 *
ANGER	0.269 **	0.256 **
SEEKING	−0.122 **	−0.123 **
CARE	−0.107 *	−0.140 **
FEAR	0.310 **	0.287 **
SADNESS	0.243 **	0.226 **
LUST	−0.163 **	−0.183 **

**Correlation is significant at the 0.01 level, *Correlation is significant at the 0.05 level.

### Multiple hierarchical regression

In the following models, primary emotions, including DISGUST-5, were incorporated as predictors, with each of the Big Five personality traits serving as a criterion. Age and sex were included as control variables.

#### Extraversion

Age and sex, when considered alone, demonstrated a minimal model fit (*F*(2, 465) = 3.499, *p* < 0.05), accounting for only 1.1% of the variance. Age emerged as a significant predictor (*t*(465) = 2.307, *β* = 0.107, *p* < 0.05), whereas sex did not contribute to the association (*t*(465) = 1.628, *β* = 0.076, *p* = 0.104).

When primary emotions, along with age and sex, were included, the model significantly associated extraversion (*F*(10, 457) = 23.264, *p* < 0.001), explaining 32.3% of the variance with a strong correlation (*R* = 0.581). The strongest predictor was PLAY (*t*(457) = 9.189, *β* = 0.417, *p* < 0.001), followed by SADNESS (*t*(457) = −2.587, *β* = −0.140, *p* < 0.05) and CARE (*t*(457) = 3.074, *β* = 0.139, *p* < 0.01). DISGUST contributed to the association with extraversion with a small effect (*t*(457) = −2.850, *β* = −0.119, *p* < 0.01).

#### Agreeableness

Age and sex together provided a weak variance explanation (*F*(2, 465) = 14.851, *p* < 0.001), accounting for 5.5% of the variance. Age had a small but significant effect on the criterion (*t*(465) = 5.400, *β* = 0.246, *p* < 0.001), while sex was not a significant predictor (*t*(465) = 0.791, *β* = 0.036, *p* = 0.429).

With the inclusion of primary emotions, the model showed a moderate variance explanation (*F*(10, 457) = 21.397, *p* < 0.001), explaining 30.4% of the variance with a strong correlation (*R* = 0.565). ANGER (*t*(457) = −6.696, *β* = −0.286, *p* < 0.001) and CARE (*t*(457) = 5.805, *β* = 0.268, *p* < 0.001) were the most influential predictors. DISGUST did not significantly contribute to the association with agreeableness (*t*(457) = −1.118, *β* = −0.048, *p* = 0.264).

#### Conscientiousness

Age and sex alone were not significant predictors of conscientiousness (*F*(2, 465) = 2.777, *p* = 0.063).

However, when primary emotions were included, the model became significant (*F*(10, 457) = 6.786, *p* < 0.001), explaining 11% of the variance. DISGUST did not independently associate conscientiousness (*t*(457) = 0.739, *β* = 0.036, *p* = 0.460). The strongest predictor was SADNESS, which had a small effect (*t*(457) = −4.483, *β* = −0.277, *p* < 0.001).

#### Neuroticism

The highest model fit was observed between primary emotions and neuroticism (*F*(10, 457) = 104.498, *p* < 0.001), explaining 68.9% of the variance with a strong correlation (*R* = 0.834). FEAR had a moderate effect (*t*(457) = 12.618, *β* = 0.479, *p* < 0.001), followed by SADNESS, which had a small effect (*t*(457) = 7.843, *β* = 0.287, *p* < 0.001). DISGUST also contributed with a small effect (*t*(457) = 4.185, *β* = 0.119, *p* < 0.001).

Age and sex, when analyzed separately, accounted for a small variance explanation (*F*(2, 465) = 35.219, *p* < 0.001), explaining 12.8% of the variance in neuroticism. Both age and sex were significant predictors (*t_age_*(465) = −7.045, *β_age_* = −0.308, *p_age_* < 0.001; *t_sex_*(465) = 3.444, *β_sex_* = 0.151, *p_sex_* < 0.01).

#### Openness

Primary emotions, along with age and sex, explained 22.9% of the variance, indicating a moderate model fit (*F*(10, 457) = 5.552, *p* < 0.001). SEEK was the strongest predictor, with a moderate effect (*t*(457) = 9.351, *β* = 0.396, *p* < 0.001). DISGUST did not significantly contribute to openness (*t*(457) = 1.157, *β* = 0.052, *p* = 0.248).

Age and sex were not significant predictors in the overall model and could not independently associate openness (*F*(2, 465) = 2.695, *p* = 0.069).

## Discussion

The aim of this study was to develop a questionnaire that captures DISGUST as a affective disposition, as there is currently no suitable assessment tool that measures disgust in the field of affective neuroscience. Additionally, the study aimed to explore how DISGUST influences human personality and if it is associated with psychiatric disorders. Despite ongoing debate about whether disgust should be recognized as a new, eighth primary emotion, it was decided, after careful consideration (as outlined in the introduction), to regard DISGUST as a primary emotion (see for further Tolchinsky et al., 2024 discussion). Consequently, a questionnaire with eight items (DISGUST-8) was created, as well as a short version with five items (DISGUST-5). Factor analyses indicated a unidimensional model, and both versions demonstrated excellent model fit. Both scales achieved high internal consistency using Cronbach’s *α* and McDonald’s *ω*, indicating that they are reliable.

Consistent with existing literature, a moderate relationship was found between DISGUST and Neuroticism. The higher the level of Neuroticism, the higher the level of DISGUST. This finding aligns with the idea that individuals with high levels of Neuroticism tend to experience negative emotions, such as DISGUST (McCrae and Costa, 2008; Montag and Panksepp, 2017). Extraversion and Agreeableness showed weak negative correlations with DISGUST. Interestingly, contrary to previous studies, no relationship was found between DISGUST and Openness or Conscientiousness. Possible reasons for these inconsistent results could be the use of different personality models (HEXACO vs. BIG-5) or the use of different disgust constructs (e.g., pathogen, core, sexual, or moral disgust) in the analysis.

Another main focus of this study was to investigate the extent to which primary emotions can associate with personality traits, and in particular, whether DISGUST contributes independently to these associations. Although regression analyses were performed, due to the cross-sectional nature of the study, these associations cannot be interpreted as causal predictions, but rather as correlational associations. The strongest associative power was found for Neuroticism, with the primary emotions explaining 68.9% of the variance. FEAR and SADNESS had the largest independent contributions, showing moderate and small effects, respectively. DISGUST also contributed with a small effect. These results are consistent with a study by Davis et al. (2003), who found that FEAR, SADNESS, and ANGER have the strongest negative associations with Neuroticism. In our model, however, ANGER did not independently associate with Neuroticism. This may be because Neuroticism is more closely associated with anxious and depressive tendencies (Watson and Clark, 1984). A strong model fit was also observed for Extraversion, with PLAY and SADNESS emerging as independent associates. DISGUST again contributed with a small effect. DISGUST did not independently associate with Agreeableness, Conscientiousness or Openness. Nevertheless, all models demonstrated weak to strong explanatory power for the Big Five personality traits, with the weakest model being the one associating with Conscientiousness. The results reaffirm that primary emotions are interwoven with personality. Remarkably, the emotions with the highest independent associative power are the same primary emotions that were most strongly correlated with the respective personality traits in Davis et al. (2003).

As previously reported, another essential feature of primary emotions is their psychiatric relevance. DISGUST correlated with all scales of the ISR. Although the associations were generally weak, the anxiety scale and the ISR sum score showed the strongest relationships with both versions of the DISGUST scale. The association with Anxiety makes sense given that DISGUST is closely linked to Neuroticism as it is widely known that individuals with high levels of Neuroticism are more prone to anxiety and worry (McCrae and Costa, 2008). Additionally, prior research has shown that disgust is involved in anxiety-related disorders (Davey, 2011; Badour and Feldner, 2016, 2018). In the case of eating disorders, individuals often report increased disgust towards themselves (Schöttke et al., 2006; Bektas et al., 2022) as well as towards food and the act of eating (Davey, 2011; Bektas et al., 2022). This could explain the association between DISGUST and Eating Disorders. Surprisingly, the association between DISGUST and Compulsion was one of the weakest, despite the fact that OCD with contamination fear is considered one of the psychiatric disorders most strongly associated with disgust (Tolchinsky et al., 2024). Since OCD is a multifaceted disorder and the questionnaire used in this study is a rather short screening tool for general compulsion, it is likely that specific sub-facets of this disorder, particularly OCD with contamination fear, are insufficiently captured by the ISR.

## Limitations and future perspectives

The results suggest that DISGUST might be usefully considered a primary emotion. Nevertheless, further studies are needed to confirm this, particularly with a more diverse sample to examine cross-cultural effects. This study primarily involved German-speaking participants with a relatively high educational background. Therefore, it seems important to adapt the questionnaire into English. The English translation of the items in this study was only for comprehension purposes and was not scientifically validated. Making the questionnaire available in English could increase its reach and enhance the study’s generalizability. It is also important to test the replicability of these findings, as the instruments used rely on self-assessment. Therefore, various biases (response tendencies, social desirability, etc.) cannot be ruled out.

The weak correlations between psychiatric symptoms and DISGUST-5 or DISGUST-8 suggest that further studies using more suitable and differentiated measurement instruments are necessary. OCD with contamination fear should be a particular focus.

Another important aspect for future research is the exploration of how different operationalizations of the Big Five and disgust constructs might influence the observed relationships to DISGUST. Furthermore, more research will be needed to investigate the convergent validity of the DISGUST scale regarding established measures of trait disgust.

Finally, for future studies it will be interesting to explore the interrelationships between DISGUST and other primary emotions by using psychometric network analysis.

## Conclusion

In conclusion, the results lend initial support for the reliability and validity of a questionnaire that captures DISGUST as a unidimensional model. DISGUST appears to have psychiatric relevance and is associated with human personality. In particular, Neuroticism and Extraversion can be associated with DISGUST. With this study, we hope to contribute to the ongoing debate on whether DISGUST should be considered a primary emotion and to provide a first questionnaire that captures DISGUST as such.

## Data Availability

The raw data supporting the conclusions of this article will be made available by the authors, without undue reservation.
