# Supplementary material for: The development and initial findings of a DISGUST scale
Source: Front Hum Neurosci. 2025 Jul 16;19:1607506. doi: 10.3389/fnhum.2025.1607506 (PMC12307371; doi:10.3389/fnhum.2025.1607506)
Supplement: Supplementary file 1 [file Data_Sheet_1.docx]

Supplementary file

Scree Plot

**Figure 4**
Scree Plot DISGUST-8


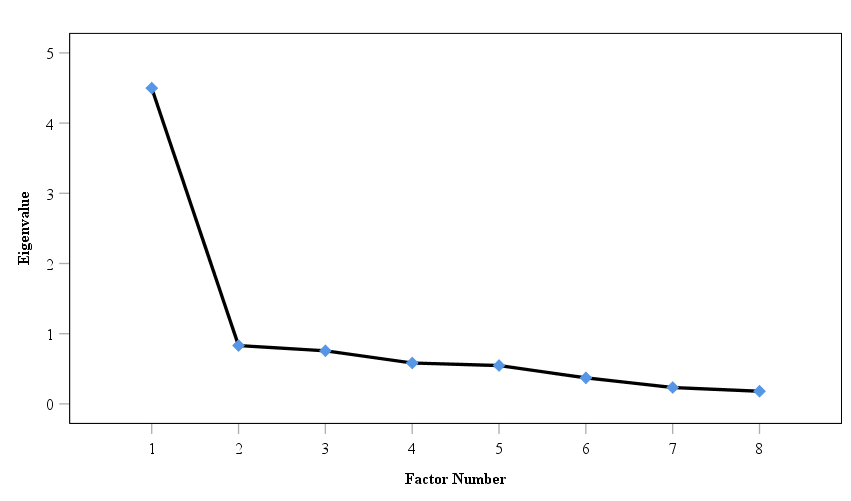


**Figure 5**
Scree Plot DISGUST-5


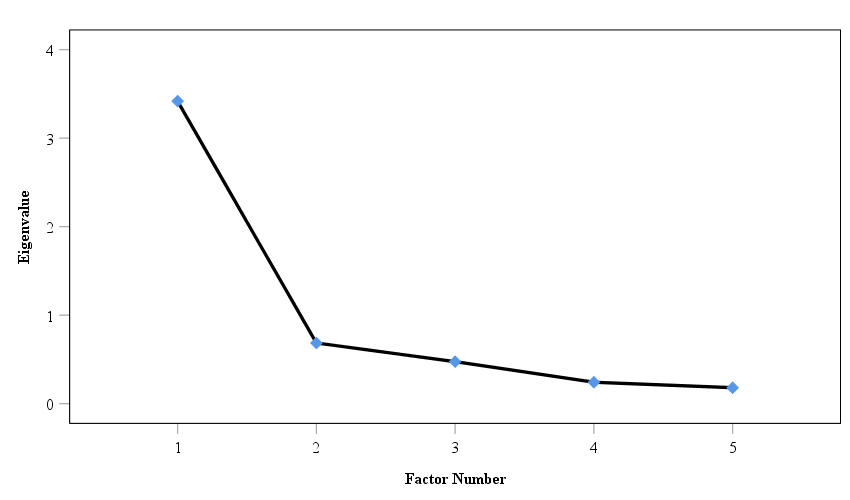


Questionnaires

***Translated version of the exploratory questionnaire: DISGUST scale***

| 1 | 2 | 3 | 4 | 5 |
| --- | --- | --- | --- | --- |
| Strongly Disagree | Disagree | Neither Agree nor Disagree | Agree | Strongly Agree |

| EK1. The smell of vomit makes me feel nauseous. | 1 | 2 | 3 | 4 | 5 |
| --- | --- | --- | --- | --- | --- |
| EK2. The mere sight of spoiled food disgusts me. | 1 | 2 | 3 | 4 | 5 |
| EK3. The sound of people vomiting repulses me. | 1 | 2 | 3 | 4 | 5 |
| EK4. Looking at open flesh wounds makes me feel lightheaded. | 1 | 2 | 3 | 4 | 5 |
| EK5. I am a person who often feels disgusted. | 1 | 2 | 3 | 4 | 5 |
| EK6. I feel disgusted by food that has already been bitten into. | 1 | 2 | 3 | 4 | 5 |
| EK7. I avoid sitting next to people with poor personal hygiene. | 1 | 2 | 3 | 4 | 5 |
| EK8. I cannot bring myself to lie in a hotel bed with dirty sheets. | 1 | 2 | 3 | 4 | 5 |
| EK9. I do not shake hands with people who do not wash their hands after using the toilet. | 1 | 2 | 3 | 4 | 5 |
| EK10. The smell of decaying animals triggers my gag reflex. | 1 | 2 | 3 | 4 | 5 |
| EK11. The smell of public toilets makes me retch. | 1 | 2 | 3 | 4 | 5 |
| EK12. The sensation of warm blood running down my skin makes me feel uncomfortable. | 1 | 2 | 3 | 4 | 5 |
| EK13. The sound of breaking bones disgusts me. | 1 | 2 | 3 | 4 | 5 |
| EK14. The thought of stepping barefoot on a snail disgusts me. | 1 | 2 | 3 | 4 | 5 |
| EK15. Bad-smelling people quickly disgust me. | 1 | 2 | 3 | 4 | 5 |
| EK16. Unpleasant odors easily trigger my gag reflex. | 1 | 2 | 3 | 4 | 5 |
| EK17. I am a person who gets disgusted quickly. | 1 | 2 | 3 | 4 | 5 |
| EK18. The thought of using someone else's toothbrush disgusts me. | 1 | 2 | 3 | 4 | 5 |
| EK19. The thought of bedbugs in my bed disgusts me. | 1 | 2 | 3 | 4 | 5 |
| EK20. The smell of decay repulses me. | 1 | 2 | 3 | 4 | 5 |
| EK21. I will no longer drink from a glass into which an insect has flown. | 1 | 2 | 3 | 4 | 5 |
| EK22. The sight of medical specimens disgusts me intensely. | 1 | 2 | 3 | 4 | 5 |
| EK23. It disgusts me when a friend offers me chocolate that looks like dog feces. | 1 | 2 | 3 | 4 | 5 |
| EK24. I cannot imagine touching a dead body. | 1 | 2 | 3 | 4 | 5 |
| EK25. The thought of seeing the intestines of an accident victim repulses me. | 1 | 2 | 3 | 4 | 5 |
| EK26. I cannot imagine eating raw, bloody meat. | 1 | 2 | 3 | 4 | 5 |
| EK27. I often feel disgusted by slimy substances. | 1 | 2 | 3 | 4 | 5 |
| EK28. I feel uncomfortable consuming food prepared by a person with a contagious disease. | 1 | 2 | 3 | 4 | 5 |
| EK29. Open wounds often disgust me. | 1 | 2 | 3 | 4 | 5 |
| EK30. People who blow their nose loudly quickly disgust me. | 1 | 2 | 3 | 4 | 5 |
| EK31. It bothers me to hear someone clearing phlegm from their throat. | 1 | 2 | 3 | 4 | 5 |
| EK32. Finding a hair in my soup disgusts me. | 1 | 2 | 3 | 4 | 5 |
| EK33. I am a person who frequently experiences disgust. | 1 | 2 | 3 | 4 | 5 |
| EK34. I cannot stand it when people chew loudly while eating. | 1 | 2 | 3 | 4 | 5 |
| EK35. I am someone who tends to experience intense disgust. | 1 | 2 | 3 | 4 | 5 |
| EK36. I cannot eat without washing my hands first. | 1 | 2 | 3 | 4 | 5 |

*Note.* English items have yet to be tested

***German version of the exploratory questionnaire: DISGUST scale***

| 1 | 2 | 3 | 4 | 5 |
| --- | --- | --- | --- | --- |
| Starke Ablehnung | Ablehnung | Weder Ablehnung noch Zustimmung | Zustimmung | Starke Zustimmung |

| EK1. Der Geruch von Erbrochenem führt bei mir zu Übelkeit. | 1 | 2 | 3 | 4 | 5 |
| --- | --- | --- | --- | --- | --- |
| EK2. Der bloße Anblick von verdorbenen Lebensmitteln ekelt mich. | 1 | 2 | 3 | 4 | 5 |
| EK3. Das Geräusch von sich übergebenden Personen widert mich an. | 1 | 2 | 3 | 4 | 5 |
| EK4. Das Betrachten von offenen Fleischwunden führt bei mir zu Benommenheit. | 1 | 2 | 3 | 4 | 5 |
| EK5. Ich bin ein Mensch, der sich oft ekelt. | 1 | 2 | 3 | 4 | 5 |
| EK6. Ich ekle mich vor bereits angebissenen Nahrungsmitteln. | 1 | 2 | 3 | 4 | 5 |
| EK7. Ich vermeide es, mich neben Personen mit schlechter Körperhygiene zu setzen. | 1 | 2 | 3 | 4 | 5 |
| EK8. Ich schaffe es nicht, mich in ein Hotelbett mit verdreckter Bettwäsche zu legen. | 1 | 2 | 3 | 4 | 5 |
| EK9. Personen, die sich nach dem Toilettengang nicht die Hände waschen, gebe ich nicht die Hand. | 1 | 2 | 3 | 4 | 5 |
| EK10. Der Geruch von verwesenden Tieren löst bei mir einen Würgereiz aus. | 1 | 2 | 3 | 4 | 5 |
| EK11. Der Geruch von öffentlichen Toiletten führt bei mir zu Brechreiz. | 1 | 2 | 3 | 4 | 5 |
| EK12. Das Gefühl von warmem, an mir herabfließendem Blut führt zu Unwohlsein. | 1 | 2 | 3 | 4 | 5 |
| EK13. Das Geräusch von brechenden Knochen ekelt mich. | 1 | 2 | 3 | 4 | 5 |
| EK14. Der Gedanke, barfuß auf eine Schnecke zu treten, ekelt mich. | 1 | 2 | 3 | 4 | 5 |
| EK15. Übelriechende Personen ekeln mich schnell. | 1 | 2 | 3 | 4 | 5 |
| EK16. Übler Geruch löst bei mir leicht Brechreiz aus. | 1 | 2 | 3 | 4 | 5 |
| EK17. Ich bin ein Mensch, der sich schnell ekelt. | 1 | 2 | 3 | 4 | 5 |
| EK18. Der Gedanke, eine fremde Zahnbürste zu benutzen, ekelt mich. | 1 | 2 | 3 | 4 | 5 |
| EK19. Der Gedanke an Bettwanzen in meinem Bett ekelt mich. | 1 | 2 | 3 | 4 | 5 |
| EK20. Fäulnisgeruch widert mich an. | 1 | 2 | 3 | 4 | 5 |
| EK21. Ich trinke aus keinem Glas mehr, in das ein Insekt geflogen ist. | 1 | 2 | 3 | 4 | 5 |
| EK22. Beim Anblick von medizinischen Exponaten ekelt es mich sehr stark. | 1 | 2 | 3 | 4 | 5 |
| EK23. Es ekelt mich, wenn mir ein Freund Schokolade anbietet, die wie Hundekot aussieht. | 1 | 2 | 3 | 4 | 5 |
| EK24. Ich kann es mir nicht vorstellen, einen toten Körper zu berühren. | 1 | 2 | 3 | 4 | 5 |
| EK25. Der Gedanke, die Gedärme eines Unfallopfers zu sehen, widert mich an. | 1 | 2 | 3 | 4 | 5 |
| EK26. Ich kann es mir nicht vorstellen, rohes, blutiges Fleisch zu essen. | 1 | 2 | 3 | 4 | 5 |
| EK27. Ich ekle mich oft vor schleimigen Substanzen. | 1 | 2 | 3 | 4 | 5 |
| EK28. Ich fühle mich unwohl dabei, Nahrung zu konsumieren, die von einer Person mit einer ansteckenden Krankheit. | 1 | 2 | 3 | 4 | 5 |
| EK29. Offene Wunden ekeln mich oft. | 1 | 2 | 3 | 4 | 5 |
| EK30. Personen, die laut schnäuzen, ekeln mich schnell. | 1 | 2 | 3 | 4 | 5 |
| EK31. Es stört mich, jemanden zu hören, der seinen Rachen von Schleim befreit. | 1 | 2 | 3 | 4 | 5 |
| EK32. Ein Haar in meiner Suppe ekelt mich schnell. | 1 | 2 | 3 | 4 | 5 |
| EK33. Ich bin ein Mensch, der sich häufig ekelt. | 1 | 2 | 3 | 4 | 5 |
| EK34. Ich ertrage es nicht, wenn Menschen beim Essen schmatzen. | 1 | 2 | 3 | 4 | 5 |
| EK35. Ich bin jemand, der zu intensivem Ekel neigt. | 1 | 2 | 3 | 4 | 5 |
| EK36. Ich kann kein Essen zu mir nehmen, ohne mir davor die Hände zu waschen. | 1 | 2 | 3 | 4 | 5 |

***DISGUST-8 English translation:***

| 1 | 2 | 3 | 4 | 5 |
| --- | --- | --- | --- | --- |
| Strongly Disagree | Disagree | Neither Agree nor Disagree | Agree | Strongly Agree |

| EK16. Unpleasant odor easily triggers my gag reflex. | 1 | 2 | 3 | 4 | 5 |
| --- | --- | --- | --- | --- | --- |
| EK17. I am a person who gets disgusted quickly. | 1 | 2 | 3 | 4 | 5 |
| EK27. I often feel disgusted by slimy substances. | 1 | 2 | 3 | 4 | 5 |
| EK33. I am a person who frequently experiences disgust. | 1 | 2 | 3 | 4 | 5 |
| EK35. I am someone who tends to experience intense disgust. | 1 | 2 | 3 | 4 | 5 |
| EK11. The smell of public toilets makes me retch. | 1 | 2 | 3 | 4 | 5 |
| EK15. Bad-smelling people disgust me quickly. | 1 | 2 | 3 | 4 | 5 |
| EK29. Open wounds often disgust me. | 1 | 2 | 3 | 4 | 5 |

*Note.* English Items have yet to be tested

***DISGUST-8 German Version:***

| 1 | 2 | 3 | 4 | 5 |
| --- | --- | --- | --- | --- |
| Starke Ablehnung | Ablehnung | Weder Ablehnung noch Zustimmung | Zustimmung | Starke Zustimmung |

| EK16. Übler Geruch löst bei mir leicht Brechreiz aus. | 1 | 2 | 3 | 4 | 5 |
| --- | --- | --- | --- | --- | --- |
| EK17. Ich bin ein Mensch, der sich schnell ekelt. | 1 | 2 | 3 | 4 | 5 |
| EK27. Ich ekle mich oft vor schleimigen Substanzen. | 1 | 2 | 3 | 4 | 5 |
| EK33. Ich bin ein Mensch, der sich häufig ekelt. | 1 | 2 | 3 | 4 | 5 |
| EK35. Ich bin jemand, der zu intensivem Ekel neigt. | 1 | 2 | 3 | 4 | 5 |
| EK11. Der Geruch von öffentlichen Toiletten führt bei mir zu Brechreiz. | 1 | 2 | 3 | 4 | 5 |
| EK15. Übelriechende Personen ekeln mich schnell. | 1 | 2 | 3 | 4 | 5 |
| EK29. Offene Wunden ekeln mich oft. | 1 | 2 | 3 | 4 | 5 |

***DISGUST-5 English translation:***

| 1 | 2 | 3 | 4 | 5 |
| --- | --- | --- | --- | --- |
| Strongly Disagree | Disagree | Neither Agree nor Disagree | Agree | Strongly Agree |

| EK16. Unpleasant odor easily triggers my gag reflex. | 1 | 2 | 3 | 4 | 5 |
| --- | --- | --- | --- | --- | --- |
| EK17. I am a person who gets disgusted quickly. | 1 | 2 | 3 | 4 | 5 |
| EK27. I often feel disgusted by slimy substances. | 1 | 2 | 3 | 4 | 5 |
| EK33. I am a person who frequently experiences disgust. | 1 | 2 | 3 | 4 | 5 |
| EK35. I am someone who tends to experience intense disgust. | 1 | 2 | 3 | 4 | 5 |

*Note.* English Items have yet to be tested

***DISGUST-5 German Version:***

| 1 | 2 | 3 | 4 | 5 |
| --- | --- | --- | --- | --- |
| Starke Ablehnung | Ablehnung | Weder Ablehnung noch Zustimmung | Zustimmung | Starke Zustimmung |

| EK16. Übler Geruch löst bei mir leicht Brechreiz aus. | 1 | 2 | 3 | 4 | 5 |
| --- | --- | --- | --- | --- | --- |
| EK17. Ich bin ein Mensch, der sich schnell ekelt. | 1 | 2 | 3 | 4 | 5 |
| EK27. Ich ekle mich oft vor schleimigen Substanzen. | 1 | 2 | 3 | 4 | 5 |
| EK33. Ich bin ein Mensch, der sich häufig ekelt. | 1 | 2 | 3 | 4 | 5 |
| EK35. Ich bin jemand, der zu intensivem Ekel neigt. | 1 | 2 | 3 | 4 | 5 |
